# Inhibition of COX‐2‐Mediated Arachidonic Acid Metabolism by FGF21‐PPARα Axis Alleviates Liver Steatosis

**DOI:** 10.1002/mco2.70999

**Published:** 2026-09-15

**Authors:** Guangsen Xu, Tingting Pan, Guiyun Wang, Feng Qiu, Junlin Leng, Qiyang Feng, Yongguo Xue, Ye Wang, Junwei Zheng, Huiying Lu, Bingyan Han, Suntao Wang, Jie Du, Jiaojian Lv, Baile Wang, Xuebo Pan

**Affiliations:** ^1^ School of Pharmaceutical Sciences Wenzhou Medical University University Town Wenzhou China; ^2^ Taizhou Traditional Chinese Medicine Hospital Taizhou China; ^3^ Department of Genetics and Developmental Science School of Life Sciences and Biotechnology Shanghai Jiao Tong University Shanghai China; ^4^ State Key Laboratory of Pharmaceutical Biotechnology The University of Hong Kong Hong Kong China; ^5^ Department of Medicine The University of Hong Kong Hong Kong China; ^6^ Guangdong‐Hong Kong Joint Laboratory for Metabolic Medicine The University of Hong Kong Hong Kong China; ^7^ Department of Cardiothoracic Surgery The Second Affiliated Hospital of Wenzhou Medical University Wenzhou Zhejiang China; ^8^ Department of Infectious Diseases The Sixth Affiliated Hospital of Wenzhou Medical University Lishui People's Hospital Lishui China

**Keywords:** arachidonic acid metabolism, cyclooxygenase‐2, fibroblast growth factor 21, liver steatosis, peroxisome proliferator‐activated receptor alpha

## Abstract

Metabolic dysfunction‐associated steatotic liver disease (MASLD) is associated with disturbances in arachidonic acid (AA) metabolism; however, the role and regulation of cyclooxygenase‐2 (COX‐2), a key enzyme in AA metabolism, during disease progression remain incompletely understood. Here, we identify fibroblast growth factor 21 (FGF21) as a previously unrecognized suppressor of hepatic COX‐2 in MASLD. Although both FGF21 and COX‐2 were elevated in steatotic livers, FGF21 deficiency further augmented hepatic COX‐2 expression in mice fed with a high‐fat diet, leading to dysregulated AA metabolism, excessive prostaglandin E_2_ (PGE_2_) production, aggravated inflammation, oxidative stress, and fibrosis. Conversely, adeno‐associated virus (AAV)‐mediated restoration of FGF21 normalized COX‐derived AA metabolites, suppressed COX‐2 activation, and ameliorated hepatic injury. Pharmacological inhibition of COX‐2 partially recapitulated the protective effects of FGF21 but failed to reverse fibrosis, suggesting that COX‐2 contributes to disease progression but is not sufficient to account for fibrosis. Mechanistically, FGF21 suppressed COX‐2 through a PPARα‐dependent pathway, whereas ERK1/2 phosphorylation acted upstream of the FGF21/PPARα axis. In patients with MASLD, circulating PGE_2_ was positively associated with liver injury after adjustment for body mass index and hepatic steatosis. Collectively, the FGF21‐PPARα‐COX‐2 axis represents a critical regulator of AA metabolic remodeling and a potential therapeutic target for MASLD.

## Graphical abstract

Under physiological conditions, the FGF21‐PPARα axis restrains COX‐2 and maintains arachidonic acid (AA) metabolic homeostasis. During steatotic liver disease, ERK1/2 phosphorylation increases alongside adaptive FGF21/PPARα activation, which is insufficient to suppress COX‐2, leading to COX‐driven AA remodeling, PGE_2_ accumulation, inflammation, and oxidative stress. ERK1/2 inhibition enhances FGF21/PPARα signaling and suppresses COX‐2‐mediated AA metabolic dysregulation.

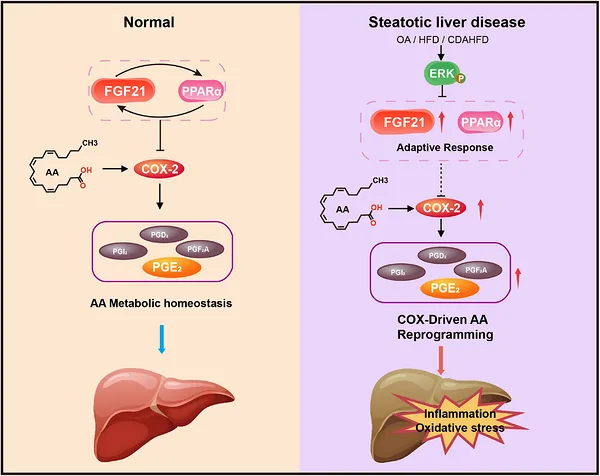

## 1 Introduction

Metabolic dysfunction‐associated steatotic liver disease (MASLD) is a prevalent chronic liver disease, affecting approximately 30% of the global population, and is characterized by hepatic lipid accumulation associated with metabolic dysfunction in the absence of significant alcohol consumption [1]. Hepatic steatosis represents an early and potentially reversible stage of MASLD. If left unmanaged, MASLD may progress from steatosis to metabolic dysfunction‐associated steatohepatitis (MASH), fibrosis, cirrhosis, and even hepatocellular carcinoma, while also increasing the risk of cardiovascular disease and type 2 diabetes [1]. Therefore, early intervention during the reversible steatosis stage, particularly through lifestyle modifications such as dietary changes and exercise, is essential to prevent disease progression and improve long‐term outcomes [2].

Hepatic steatosis is associated with alterations in polyunsaturated fatty acids (PUFAs) composition, most notably arachidonic acid (AA), an omega‐6 PUFA, and precursor for numerous bioactive lipid mediators [3]. Accumulating evidence implicates AA metabolism in the early progression of steatotic liver disease, with several AA‐derived metabolites emerging as candidate indicators of advancing liver injury [3]. AA is metabolized through multiple enzymatic cascades—the cyclooxygenase (COX), lipoxygenase (LOX), and cytochrome P450 (CYP) pathways—each generating distinct bioactive lipid mediators that orchestrate hepatic inflammation and tissue remodeling. Among these pathways, increasing evidence indicates that COX‐derived metabolites are particularly relevant to the progression of liver steatosis.

Cyclooxygenase‐2 (COX‐2) is a key enzyme in AA metabolism that converts AA into prostaglandins, most prominently prostaglandin E_2_ (PGE_2_), a major mediator of inflammation and fibrosis during hepatic steatosis. Increased hepatic COX‐2 expression has been reported in high‐fat diet (HFD)‐induced liver injury and is supported by evidence from both human studies and animal models showing enhanced inflammation, reactive oxygen species (ROS) production, lipid peroxidation, and extracellular matrix deposition [4]. Consistently, pharmacological inhibition of COX‐2 attenuates inflammatory cytokine production, oxidative stress, and fibrotic responses in steatotic livers, supporting a pathogenic role for COX‐2/PGE_2_ signaling. In addition, interactions between COX‐2‐derived prostaglandins and other AA metabolites, such as hydroxyeicosatetraenoic acids (HETEs), may further exacerbate early hepatic steatosis [5−7]. However, the upstream mechanisms governing COX‐2 activation in HFD‐induced steatosis remain poorly defined.

Fibroblast growth factor 21 (FGF21), a hepatokine secreted predominantly by the liver, exerts pleiotropic metabolic effects and has been shown in preclinical models to ameliorate hepatic steatosis, enhance insulin sensitivity, and reduce inflammation and fibrosis [8, 9]. Genetic or pharmacological activation of FGF21 signaling decreases hepatic lipid accumulation and alleviates inflammation and fibrosis, with PPARα serving as a key intermediary in FGF21‐mediated metabolic regulation [10, 11]. Notably, PPARα has also been implicated in the regulation of COX‐2 expression, raising the possibility that PPARα may provide a mechanistic link between FGF21 signaling and COX‐2‐mediated AA metabolism [12]. Given the established role of COX‐2‐mediated AA metabolism in steatotic liver injury, these observations raise the possibility that FGF21 may counteract HFD‐induced hepatic steatosis by modulating COX‐2/PGE_2_ signaling through PPARα. We therefore hypothesize that FGF21‐PPARα signaling suppresses COX‐2‐mediated AA metabolism, thereby providing a new mechanism to counteract HFD‐induced hepatic steatosis.

In this study, we performed clinical correlation analyses to investigate the associations among circulating FGF21, PGE_2_, and liver injury in patients with MASLD, and employed targeted metabolomic profiling to examine the role of FGF21 in COX‐2‐associated AA metabolism. Using wild‐type (WT) and FGF21 knockout (FGF21KO) mice, together with AAV‐mediated FGF21 restoration in HFD‐ and CDAHFD (choline‐deficient, L‐amino‐acid‐defined high‐fat diet)‐induced models, we systematically evaluated the effects of FGF21 on hepatic COX‐2 activation, AA metabolic remodeling, and the associated inflammatory and fibrotic responses. We further assessed whether pharmacological inhibition of COX‐2 recapitulates the protective effects of FGF21, and elucidated the respective contributions of the FGF21/PPARα axis and ERK1/2 signaling to COX‐2 regulation.

## 2 Results

### 2.1 Associations of Circulating FGF21 and Circulating PGE_2_ With Liver Injury in Patients With MASLD

A total of 57 participants were initially enrolled, three of whom were subsequently excluded for not meeting the inclusion criteria. The baseline demographic and clinical characteristics of the remaining study population are summarized in Table S1. Compared with healthy controls, patients with MASLD exhibited significantly higher body mass index (BMI; 21.95 ± 2.48 vs. 26.35 ± 3.58 kg/m^2^; *p* < 0.0001), total cholesterol (4.66 ± 0.66 vs. 5.56 ± 1.05 mmol/L; *p* = 0.0005), low‐density lipoprotein cholesterol (2.60 ± 0.60 vs. 3.04 ± 0.51 mmol/L; *p* = 0.0043), triglycerides (1.18 ± 0.60 vs. 2.41 ± 1.56 mmol/L; *p* = 0.0006), fasting plasma glucose (5.07 ± 0.57 vs. 6.10 ± 1.64 mmol/L; *p* = 0.0050), controlled attenuation parameter (CAP; 205.71 ± 21.49 vs. 299.79 ± 40.14 dB/m; *p* < 0.0001), and liver stiffness measurement (LSM; 5.05 ± 1.05 vs. 8.02 ± 3.58 kPa; *p* = 0.0002). Age did not differ significantly between the two groups.

**FIGURE 1:**
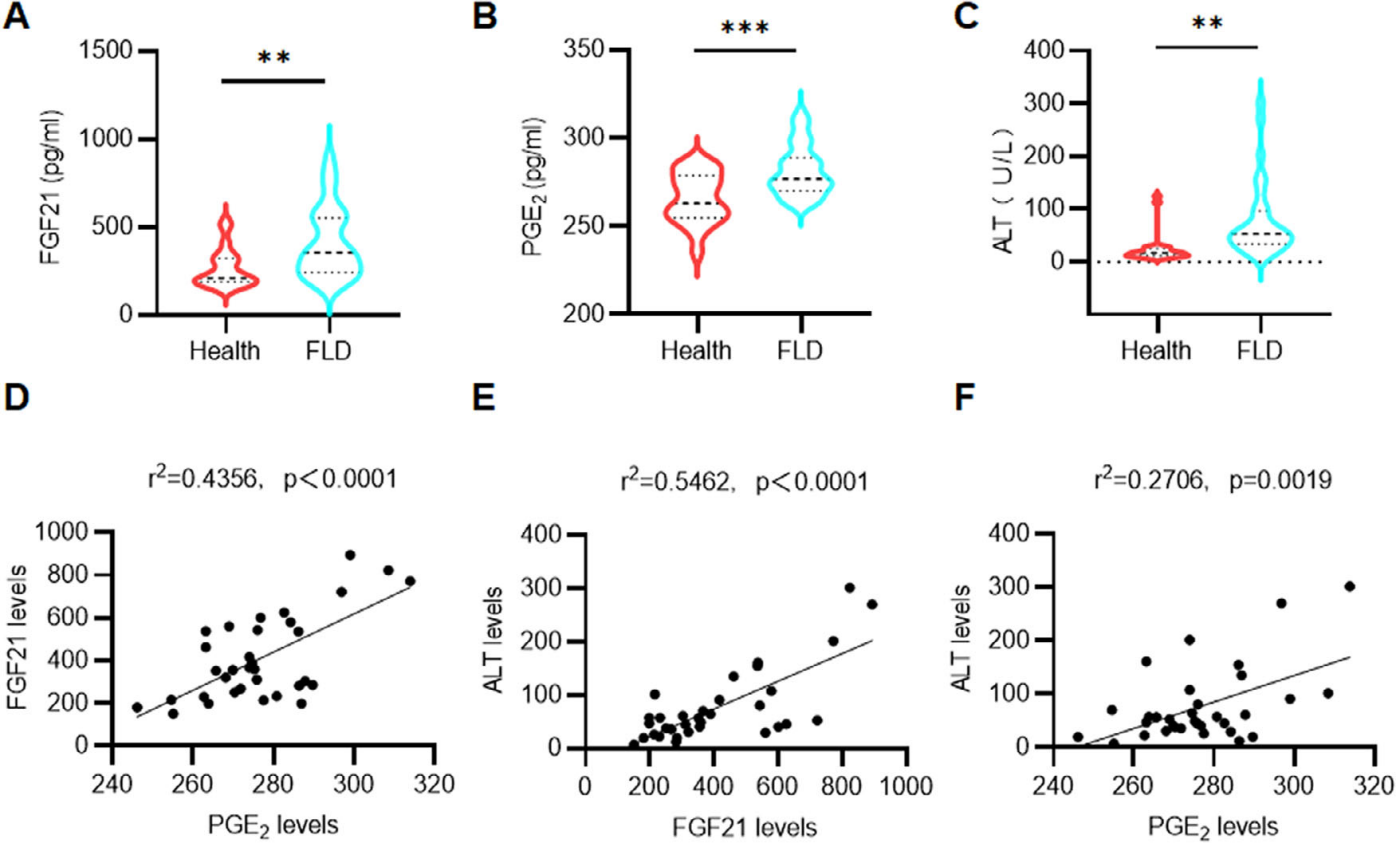
Serum FGF21, PGE_2_, and ALT levels and their correlations in patients with MASLD. Data were obtained from patients with MASLD (*n* = 33) and healthy controls (*n* = 21). (A) Serum FGF21 levels. (B) Serum PGE_2_ levels. (C) Serum ALT levels. (D) Correlation between serum FGF21 and PGE_2_ in patients with MASLD. (E) Correlation between serum FGF21 and ALT in patients with MASLD. (F) Correlation between serum PGE_2_ and ALT in patients with MASLD. For panels (A)–(C), differences between groups were analyzed by unpaired two‐tailed Student's *t*‐test; for panels (D)–(F), correlations were assessed by Pearson's correlation analysis. Violin plots depict the distribution of values, with dashed lines indicating the median and interquartile range. Data are presented as mean ± SEM. **p < 0.01, ***p < 0.001.

Because COX‐2 is not a secretory protein and cannot be directly measured in circulation, we used serum PGE_2_, a major downstream product of AA metabolism generated through COX‐dependent pathways, as a surrogate marker of COX‐2‐associated hepatic inflammatory activity. Although circulating PGE_2_ may derive from both hepatic and extrahepatic sources, serum levels may partially reflect hepatic COX‐related metabolic alterations in MASLD. As illustrated in Figure 1A–C, serum FGF21, PGE_2_, and alanine aminotransferase (ALT) levels were all significantly elevated in patients with MASLD relative to healthy controls. Correlation analyses further revealed positive associations between serum FGF21 and PGE_2_ levels (*r* ^2^ = 0.4356, *p* < 0.0001; Figure 1D), serum FGF21 and ALT levels (*r* ^2^ = 0.5462, *p* < 0.0001; Figure 1E), and serum PGE_2_ and ALT levels (*r* ^2^ = 0.2706, *p* = 0.0019; Figure 1F).

Given that BMI and CAP differed significantly between the two groups and may reflect metabolic status and hepatic steatosis severity, partial correlation analyses were performed with adjustment for these variables. After controlling for BMI and CAP, the associations of FGF21 with PGE_2_ (partial *r* = 0.205, *p* = 0.137) and ALT (partial *r* = 0.135, *p* = 0.331) were attenuated and no longer reached statistical significant (Figure S1A,B). In contrast, the positive correlation between PGE_2_ and ALT remained significant after adjustment (partial *r* = 0.409, *p* = 0.0022) (Figure S1C). These findings indicate that circulating FGF21 levels are influenced by metabolic status and hepatic steatosis severity in patients with MASLD. Nonetheless, the observed association between FGF21 and PGE_2_ in the clinical cohort prompted us to investigate the relationship between FGF21 and COX‐2 signaling in subsequent experimental studies.

### 2.2 FGF21 Deficiency Aggravates HFD‐Induced Hepatic Injury and COX‐2‐Related AA Metabolic Disturbance

**FIGURE 2:**
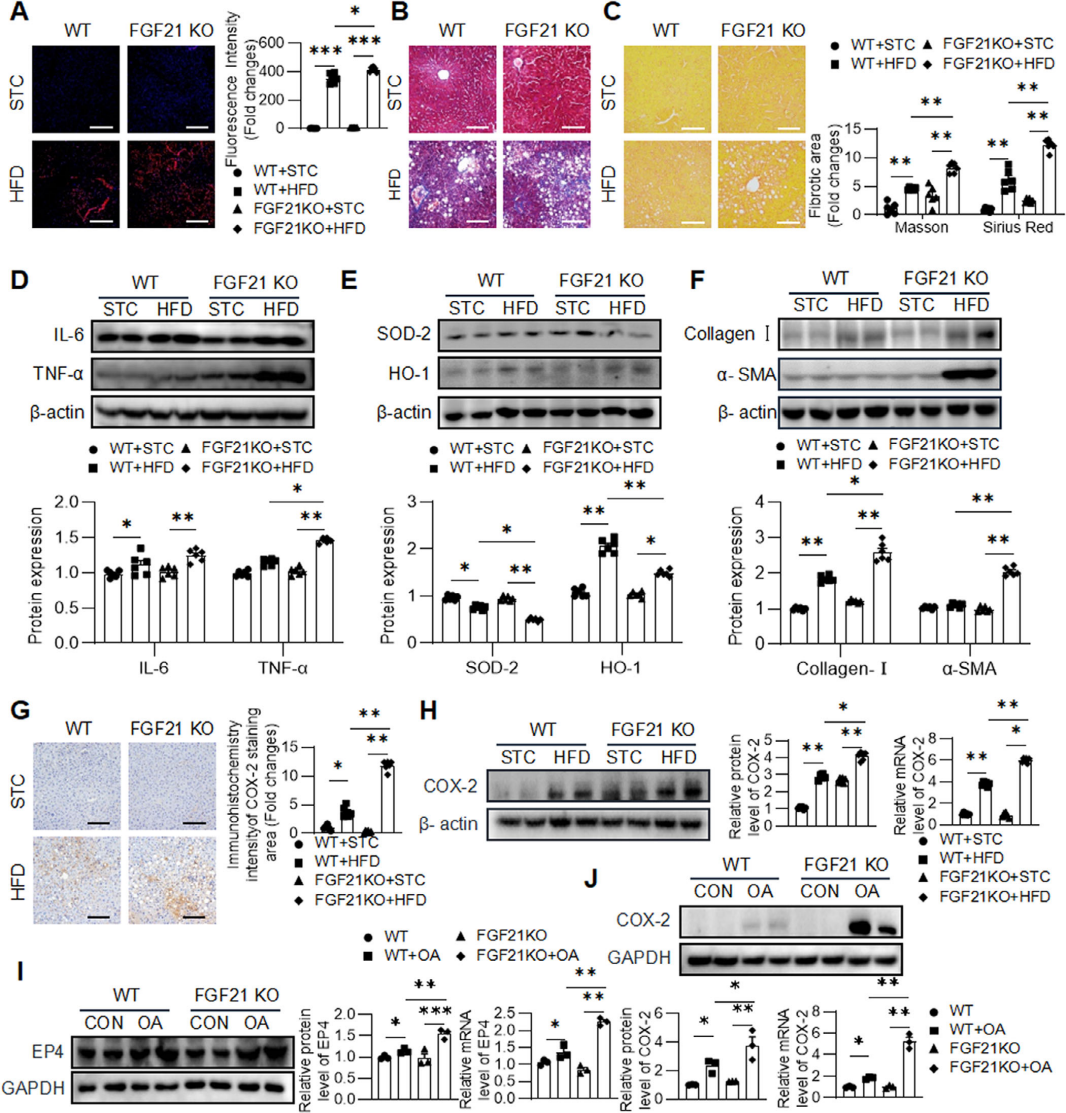
FGF21 deficiency aggravates hepatic inflammation, oxidative stress, and fibrosis through activation of the COX‐2/PGE_2_/EP4 axis. (A) Hepatic ROS production assessed by DHE staining. (B) and (C) Hepatic fibrosis in the four experimental groups detected by Masson staining and Sirius red staining, respectively. Scale bar, 50 µm. (D) Hepatic protein levels of IL‐6 and TNF‐α determined by western blotting. (E) Hepatic protein levels of SOD2 and HO‐1 in the indicated mice determined by western blotting. (F) Hepatic protein levels of Collagen I and α‐SMA determined by western blotting. (G) Representative immunohistochemical staining for COX‐2 in liver tissue. Scale bar, 50 µm. (H) Hepatic COX‐2 protein and mRNA levels determined by western blotting and qRT‐PCR, respectively. (I and J) Protein and mRNA levels of EP4 and COX‐2 in primary hepatocytes isolated from WT and FGF21 mice, with or without OA treatment; protein levels were assessed by western blotting and mRNA levels by qRT‐PCR. Data are presented as mean ± SEM. For in vivo experiments (A–H), *n* = 6 per group; statistical comparisons were performed by one‐way ANOVA followed by Tukey's post hoc test. For in vitro experiments (I) and (J), results are from three independent biological replicates; statistical analysis was performed by one‐way ANOVA followed by Tukey's post hoc test; *p < 0.05, **p < 0.01, ***p < 0.001.

We next investigated whether FGF21 deficiency alters hepatic COX‐2‐associated AA metabolism during HFD‐induced hepatic steatosis. Elevated hepatic COX‐2 expression has been consistently demonstrated during the development of steatotic liver disease [13, 14]. Likewise, FGF21 expression is upregulated in steatotic livers and in the circulation of patients with MASLD, suggesting that both molecules may be induced as part of the disease response [15]. Compared with HFD‐fed WT mice, FGF21KO mice exhibited more severe hepatic injury, as evidenced by increased ROS accumulation (Figure 2A), aggravated histopathological alterations, and enhanced collagen deposition detected by Masson and Sirius Red staining (Figure 2B,C). Hepatic expression of the inflammatory cytokines IL‐6 and TNF‐α was further elevated in FGF21KO mice following HFD feeding (Figure 2D). In addition, antioxidant‐related proteins, including SOD2 and HO‐1, were markedly dysregulated (Figure 2E), whereas fibrosis‐associated proteins, including collagen I and α‐SMA, were significantly upregulated (Figure 2F). Collectively, these findings indicate that FGF21 deficiency exacerbates HFD‐induced hepatic oxidative stress, inflammation, and fibrotic remodeling.

To further characterize the systemic metabolic phenotype, we monitored body weight, fat accumulation, tissue weight distribution, liver morphology, glucose metabolism, and energy expenditure. HFD increased body weight and fat mass in both WT and FGF21KO mice, while FGF21KO mice exhibited more severe hepatic steatosis and hepatomegaly after HFD feeding (Figure S2A–E). Consistently, hepatic inflammatory markers and α‐SMA staining were further elevated in HFD‐fed FGF21KO mice (Figure S2F–I). Metabolic cage analysis revealed that FGF21 deficiency altered food intake, oxygen consumption, carbon dioxide production, and respiratory exchange ratio under HFD conditions, suggesting impaired systemic energy metabolism and substrate utilization (Figure S3). In addition, HFD‐fed FGF21KO mice displayed worsened lipid profiles and glucose intolerance, together with elevated serum ALT and AST levels, further supporting aggravated metabolic dysfunction and liver injury attributable to FGF21 deficiency (Figure S4A–H).

**FIGURE 3:**
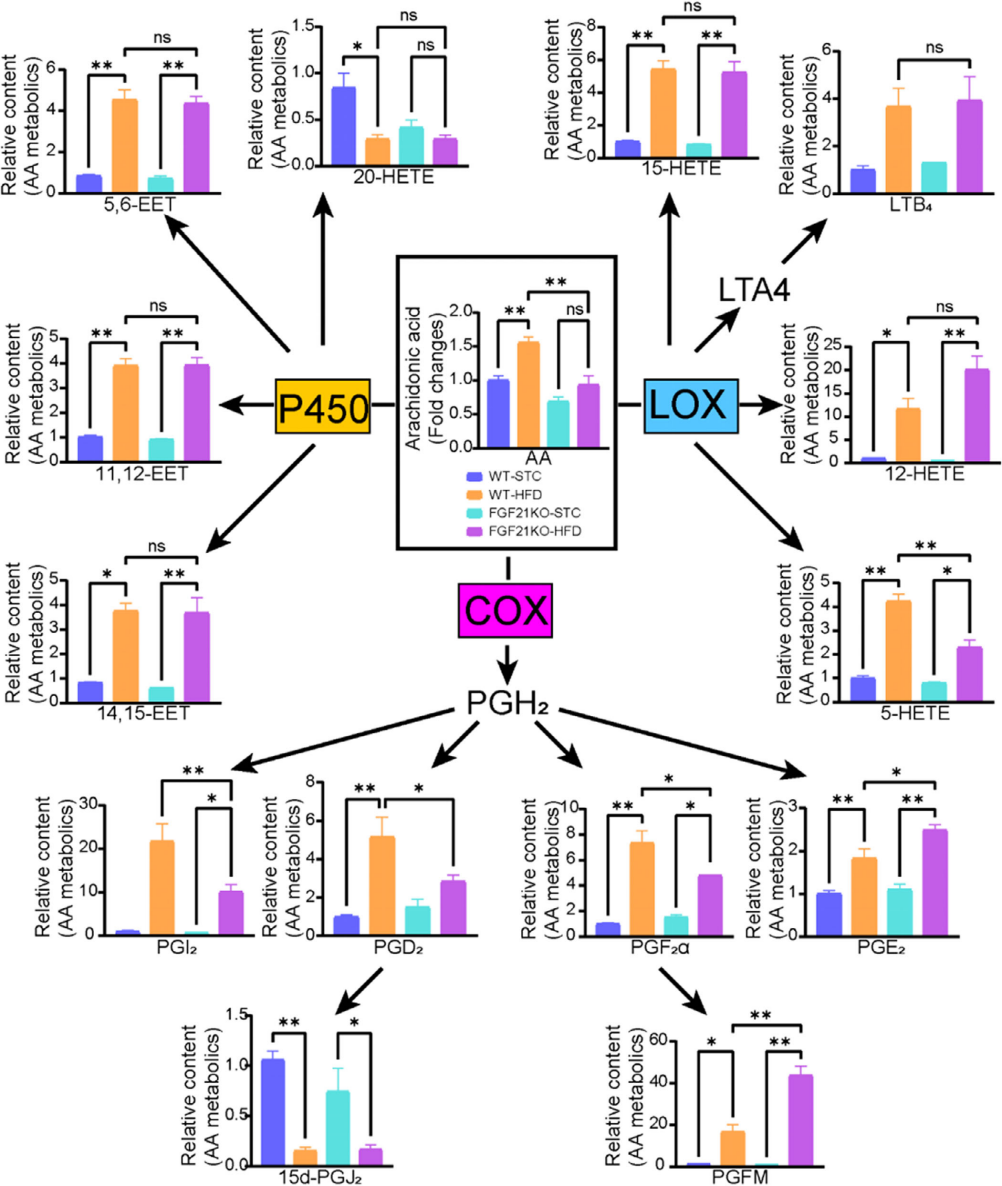
FGF21 ablation exacerbates COX‐mediated disruption of hepatic AA metabolism in HFD‐induced liver steatosis. Hepatic levels of representative metabolites across the three AA metabolic pathways were measured by LC‐MS/MS in the indicated mice: the CYP pathway (20‐HETE, 5,6‐EET, 11,12‐EET, and 14,15‐EET); the LOX pathway (15‐HETE, LTB_4_, 12‐HETE and 5‐HETE); and the COX pathway (PGI_2_, PGD_2_, PGF_2_α, PGE_2_, 15d‐PGJ_2_, and PGFM). Data are presented as mean ± SEM; *n* = 6 per group. Statistical comparisons among groups were performed by one‐way ANOVA followed by Tukey's post hoc test. *p < 0.05, **p < 0.01, ***p < 0.001.

Previous studies have documented elevated hepatic COX‐2 expression accompanied by disturbances in AA metabolism during the development of steatotic liver disease [16, 17]. Given the clinical association between PGE_2_ and ALT, and the marked inflammatory phenotype observed in FGF21KO mice, we next investigated whether FGF21 deficiency altered AA metabolism. LC‐MS/MS‐based targeted metabolomic analysis was performed to quantify AA and its downstream metabolites derived from the CYP, LOX, and COX pathways. In liver tissues, HFD feeding broadly remodeled AA metabolism, with significant changes in metabolites across the CYP, LOX, and COX pathways (Figure 3). Notably, COX‐derived metabolites were markedly disturbed in HFD‐fed FGF21KO mice. In particular, PGE_2_ and PGFM were further increased in FGF21KO mice under HFD conditions, whereas several other prostaglandin‐related metabolites showed differential regulation (Figure 3). These results suggest that FGF21 deficiency preferentially aggravates hepatic COX‐related AA metabolic disturbance during HFD‐induced liver injury. In contrast to the marked hepatic alterations, serum AA metabolites showed relatively limited changes. Several serum metabolites across the CYP, LOX, and COX pathways, including PGE_2_ and other prostaglandin‐related products, were not significantly altered between HFD‐fed WT and FGF21KO mice (Figure S5). These findings collectively indicate that the AA metabolic disturbance induced by FGF21 deficiency is more prominent in the liver than in the circulation.

We therefore examined whether the hepatic COX‐2/PGE_2_ axis was activated in FGF21KO mice. Immunohistochemical staining showed that hepatic COX‐2 expression was increased after HFD feeding and was further enhanced in FGF21KO mice (Figure 2G). Consistently, both COX‐2 protein and mRNA levels were markedly upregulated in the livers of HFD‐fed FGF21KO mice compared with HFD‐fed WT mice (Figure 2H). Moreover, the PGE_2_ receptor EP4 was also elevated in FGF21KO mice under OA stimulation, accompanied by further induction of COX‐2 expression in primary hepatocytes isolated from FGF21KO mice (Figure 2I,J). Together, these findings indicate that FGF21 deficiency aggravates HFD‐induced hepatic injury and is associated with enhanced activation of the COX‐2/PGE_2_ signaling axis.

### 2.3 FGF21 Restoration Attenuates Hepatic Injury and COX‐Related AA Metabolic Disturbance in FGF21KO Mice

**FIGURE 4:**
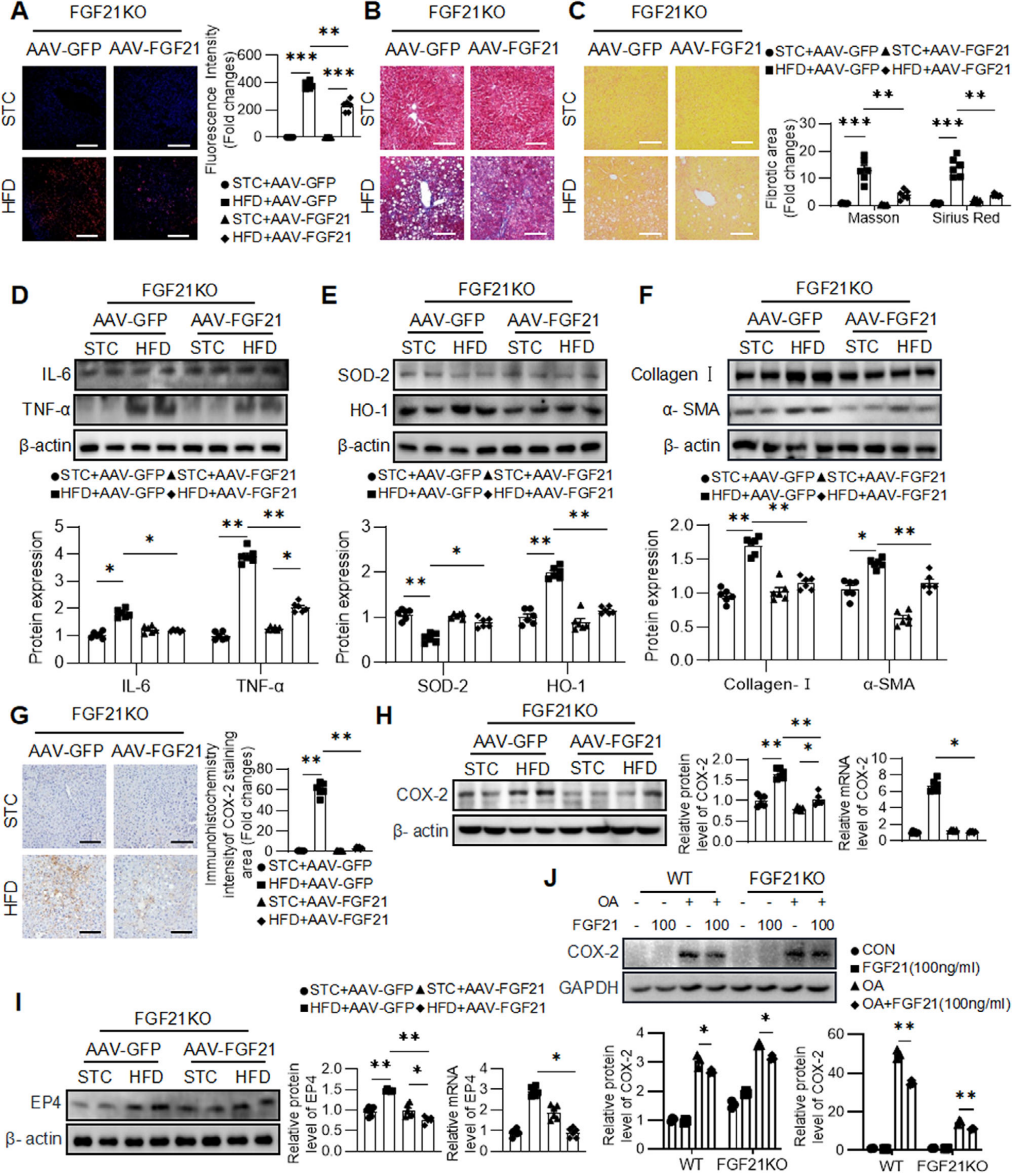
Restoration of FGF21 rescues HFD‐induced hepatic inflammation, oxidative stress, and fibrosis by suppressing the COX‐2/PGE_2_/EP4 axis. (A) Hepatic ROS production assessed by DHE staining. (B and C) Hepatic fibrosis in the four experimental groups detected by Masson's trichrome staining and Sirius Red staining, respectively. Scale bar, 50 µm. (D) Hepatic protein levels of IL‐6 and TNF‐α determined by western blotting. (E) Hepatic protein levels of SOD2 and HO‐1 levels in indicated mice determined by western blotting. (F) Hepatic protein levels of Collagen I and α‐SMA determined by western blotting. (G) Representative immunochemistry staining for COX‐2 in liver tissue. Scale bar, 50 µm. (H) Hepatic COX2 protein and mRNA levels determined by western blotting and qRT‐PCR, respectively. (I) Hepatic EP4 protein and mRNA levels in mouse liver examined by western blotting and qRT‐PCR, respectively. (J) Protein and mRNA levels of COX‐2 and EP4 in primary hepatocytes treated with OA and/or recombinant FGF21, assessed by western blotting and qRT‐PCR, respectively. Data are presented as mean ± SEM. For in vivo experiments (A–I), *n* = 6 per group; statistical comparisons were performed by one‐way ANOVA followed by Tukey's post hoc test. For in vitro experiments (J), results are from three independent biological replicates. Statistical analysis was performed by one‐way ANOVA followed by Tukey's post hoc test. *p < 0.05, **p < 0.01, ***p < 0.001.

After observing that FGF21 deficiency aggravated HFD‐induced hepatic injury and enhanced COX‐related AA metabolic disturbance, we next examined whether restoring FGF21 expression could reverse these pathological alterations. FGF21KO mice were treated with AAV‐GFP or AAV‐FGF21 and then subjected to STC or HFD feeding. AAV‐FGF21 administration markedly elevated circulating FGF21 levels in FGF21KO mice, confirming successful FGF21 supplementation (Figure S6H). Compared with HFD‐fed FGF21KO mice receiving AAV‐GFP, AAV‐FGF21‐treated mice showed attenuated HFD‐induced liver injury, as evidenced by reduced ROS accumulation, improved hepatic histology, and decreased collagen deposition detected by Masson and Sirius Red staining (Figure 4A–C). Consistently, AAV‐FGF21 reduced hepatic IL‐6 and TNF‐α expression under HFD conditions (Figure 4D). Antioxidant‐related proteins, including SOD2 and HO‐1, were partially restored after FGF21 supplementation (Figure 4E), whereas fibrosis‐associated proteins, including collagen I and α‐SMA, were markedly decreased (Figure 4F). Collectively, these results indicate that FGF21 supplementation alleviates HFD‐induced oxidative stress, inflammation, and fibrotic remodeling in FGF21KO mice.

We further assessed the metabolic phenotype after AAV‐FGF21 treatment. AAV‐FGF21 reduced HFD‐induced body weight gain and fat accumulation in FGF21KO mice (Figure S6A,B). Tissue weight analysis showed that AAV‐FGF21 decreased liver and white adipose tissue weight‐to‐body‐weight ratios under HFD conditions (Figure S6C). Gross liver morphology and H&E staining further revealed that AAV‐FGF21 markedly ameliorated hepatic steatosis and reduced hepatocellular vacuolation in HFD‐fed FGF21KO mice (Figure S6D,E). In addition, AAV‐FGF21 reduced hepatic IL‐6 and TNF‐α mRNA levels (Figure S6F,G). Consistent with these findings, AAV‐FGF21 improved lipid profiles and glucose metabolism and decreased serum ALT and AST levels in HFD‐fed FGF21KO mice (Figure S4I–P). These data further support the protective effect of FGF21 supplementation against HFD‐induced metabolic dysfunction and liver injury. To determine whether the phenotypic improvement induced by FGF21 supplementation was accompanied by restoration of AA metabolic homeostasis, targeted LC‐MS/MS analysis of hepatic AA metabolites was performed. AAV‐FGF21 partially normalized hepatic AA levels in HFD‐fed FGF21KO mice and broadly reshaped AA metabolism across CYP, LOX, and COX branches (Figure S7). Notably, FGF21 supplementation reduced HFD‐induced accumulation of several COX‐derived metabolites, including PGF_2_α, PGD_2_, and PGE_2_, while increasing 15d‐PGJ_2_ levels (Figure S7). These findings suggest that restoration of FGF21 expression alleviates HFD‐induced hepatic AA metabolic disturbance, particularly within the COX‐related prostaglandin pathway.

We next examined whether FGF21 supplementation suppresses the hepatic COX‐2/PGE_2_ signaling axis. Immunohistochemical staining showed that AAV‐FGF21 markedly reduced hepatic COX‐2 expression in HFD‐fed FGF21KO mice (Figure 4G). Consistently, AAV‐FGF21 decreased both COX‐2 protein and mRNA levels in the liver (Figure 4H). In parallel, hepatic EP4 protein and mRNA expression were also reduced after AAV‐FGF21 treatment (Figure 4I). Moreover, recombinant FGF21 treatment suppressed OA‐induced COX‐2 and EP4 expression in primary hepatocytes, an effect that was particularly pronounced in cells derived from FGF21KO mice (Figure 4J). Together, these results demonstrate that FGF21 supplementation alleviates hepatic injury and AA metabolic disturbance in FGF21KO mice, accompanied by inhibition of the hepatic COX‐2/PGE_2_ signaling axis. These findings establish a mechanistic link between FGF21 restoration and COX‐2 suppression, prompting us to assess whether pharmacological inhibition of COX‐2 recapitulates the protective effects of FGF21.

### 2.4 Pharmacological Inhibition of COX‐2 Partially Recapitulates the Protective Effects of FGF21

**FIGURE 5:**
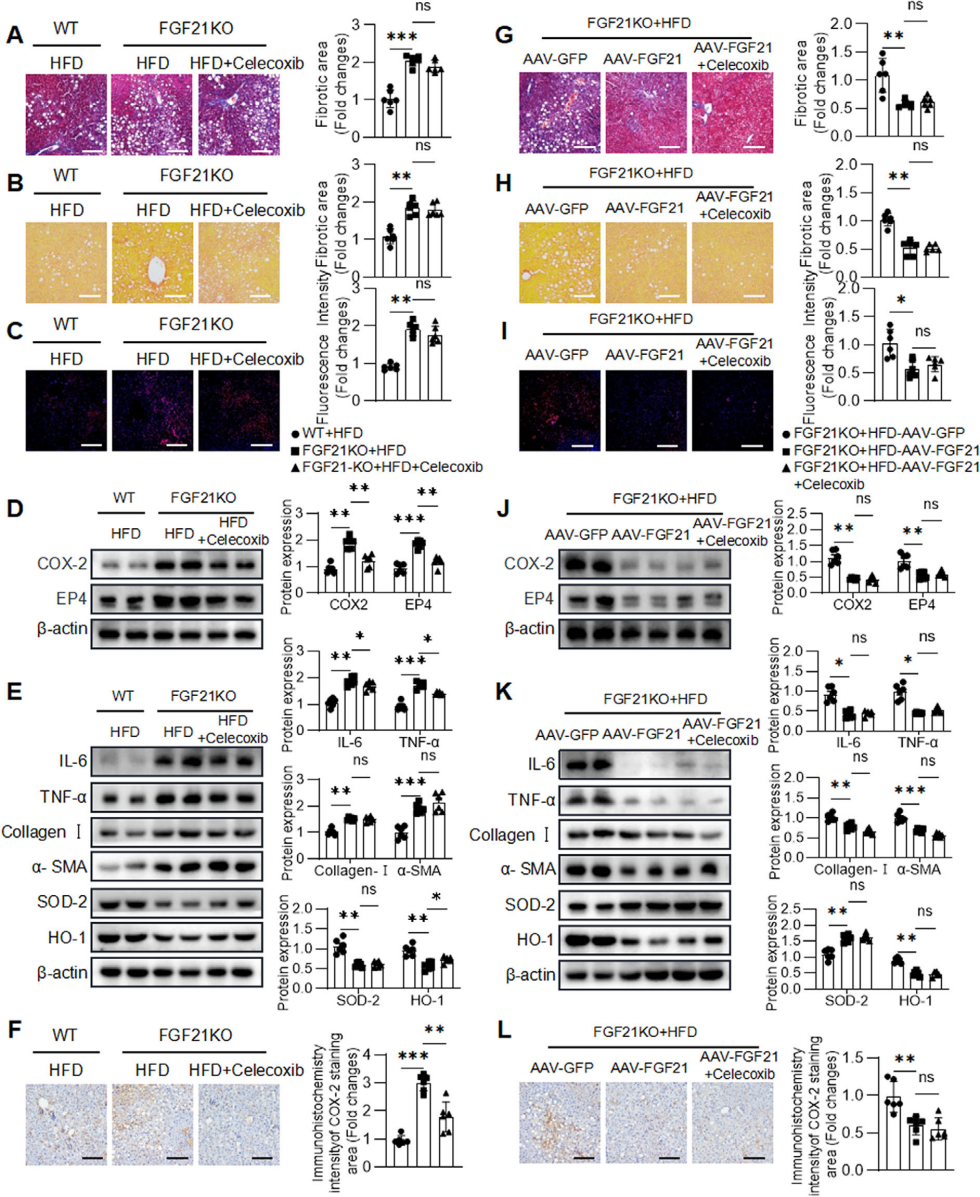
COX‐2 inhibition partially attenuates hepatic inflammation and oxidative stress in FGF21‐deficient mice but confers no additional protection following FGF21 restoration. (A and G) Representative Masson's trichrome staining of liver sections from WT+HFD, FGF21KO+HFD, FGF21KO+HFD+Celecoxib mice (A), and FGF21KO+HFD+AAV‐GFP, FGF21KO+HFD+AAV‐FGF21, FGF21KO+HFD+AAV‐FGF21+Celecoxib mice (G); quantification of fibrotic area is shown. Scale bar, 50 µm. (B and H) Representative Sirius Red staining of liver sections from the indicated groups; quantification of fibrotic area is shown. Scale bar, 50 µm. (C and I) Hepatic ROS production assessed by DHE staining in the indicated groups; quantification of fluorescence intensity is shown. Scale bar, 50 µm. (D and J) Hepatic protein levels of COX‐2 and EP4 in the indicated groups determined by western blotting; quantitative analyses of protein expression are shown. (E and K) Hepatic protein levels of IL‐6, TNF‐α, Collagen I, α‐SMA, SOD2, and HO‐1 in the indicated groups determined by western blotting; quantitative analyses are shown. (F) and (L) Representative immunohistochemical staining for COX‐2 in the indicated groups; quantification of COX‐2‐positive area is shown. Scale bar, 50 µm. Data are presented as mean ± SEM; *n* = 6 per group. Statistical comparisons among groups were performed by one‐way ANOVA followed by Tukey's post hoc test. *p < 0.05, **p < 0.01, ***p < 0.001.

To further determine whether COX‐2 contributes to the aggravated hepatic injury observed in FGF21‐deficient mice, we administered Celecoxib, a selective COX‐2 inhibitor, to HFD‐fed FGF21KO mice. Compared with HFD‐fed WT mice, FGF21KO mice displayed more severe hepatic fibrosis, oxidative stress, and inflammatory responses. Celecoxib treatment partially alleviated these pathological alterations. Specifically, Celecoxib modestly attenuated ROS accumulation in the livers of HFD‐fed FGF21KO mice (Figure 5C), accompanied by decreased hepatic COX‐2 and EP4 expression (Figure 5D,F). In addition, hepatic IL‐6 and TNF‐α levels were significantly reduced following Celecoxib administration (Figure 5E). However, Celecoxib exerted limited effects on fibrosis‐related markers, including collagen I and α‐SMA, as well as antioxidant proteins SOD2 and HO‐1 (Figure 5E). Histological assessment by Masson and Sirius Red staining likewise revealed no significant reduction in collagen deposition after Celecoxib treatment (Figure 5A,B). These findings suggest that COX‐2 contributes substantially—but not exclusively—to FGF21 deficiency‐induced liver injury.

Given that AAV‐mediated FGF21 supplementation markedly suppressed hepatic COX‐2 expression, we next investigated whether additional COX‐2 inhibition could further enhance the protective effects of FGF21. Compared with AAV‐GFP‐treated FGF21KO mice, AAV‐FGF21 administration reduced hepatic fibrosis, oxidative stress, inflammatory cytokine expression, and COX‐2/EP4 activation (Figure 5B,D,F,H,J,L). However, concomitant Celecoxib treatment failed to confer additional benefits beyond those achieved by AAV‐FGF21 alone. No further reductions were observed in hepatic collagen deposition, ROS accumulation, COX‐2/EP4 expression, inflammatory markers, fibrosis‐associated proteins, or antioxidant‐related proteins (Figure 5G–L). To further characterize the metabolic consequences of COX‐2 inhibition, gross liver morphology, hepatic steatosis, body weight, serum PGE_2_ levels, and liver function parameters were assessed (Figure S8). Celecoxib modestly improved liver appearance and reduced serum ALT and AST levels in HFD‐fed FGF21KO mice, accompanied by decreased hepatic PGE_2_ levels (Figure S8A–G). Nevertheless, Celecoxib exerted minimal additional effects in AAV‐FGF21‐treated mice, as liver morphology, steatosis, serum PGE_2_, and biochemical indices were largely comparable between the AAV‐FGF21 and AAV‐FGF21 plus Celecoxib groups (Figure S8H–N).

Collectively, these findings indicate that pharmacological inhibition of COX‐2 partially attenuates hepatic inflammation and oxidative stress in FGF21‐deficient mice but is insufficient to fully reverse hepatic fibrosis. Moreover, the lack of additive effects between Celecoxib and FGF21 supplementation suggests that suppression of the COX‐2/PGE_2_ axis represents a key mechanism underlying the hepatoprotective actions of FGF21.

### 2.5 The FGF21‐COX‐2 Axis Is Reproducible Across Distinct MASLD Models

**FIGURE 6:**
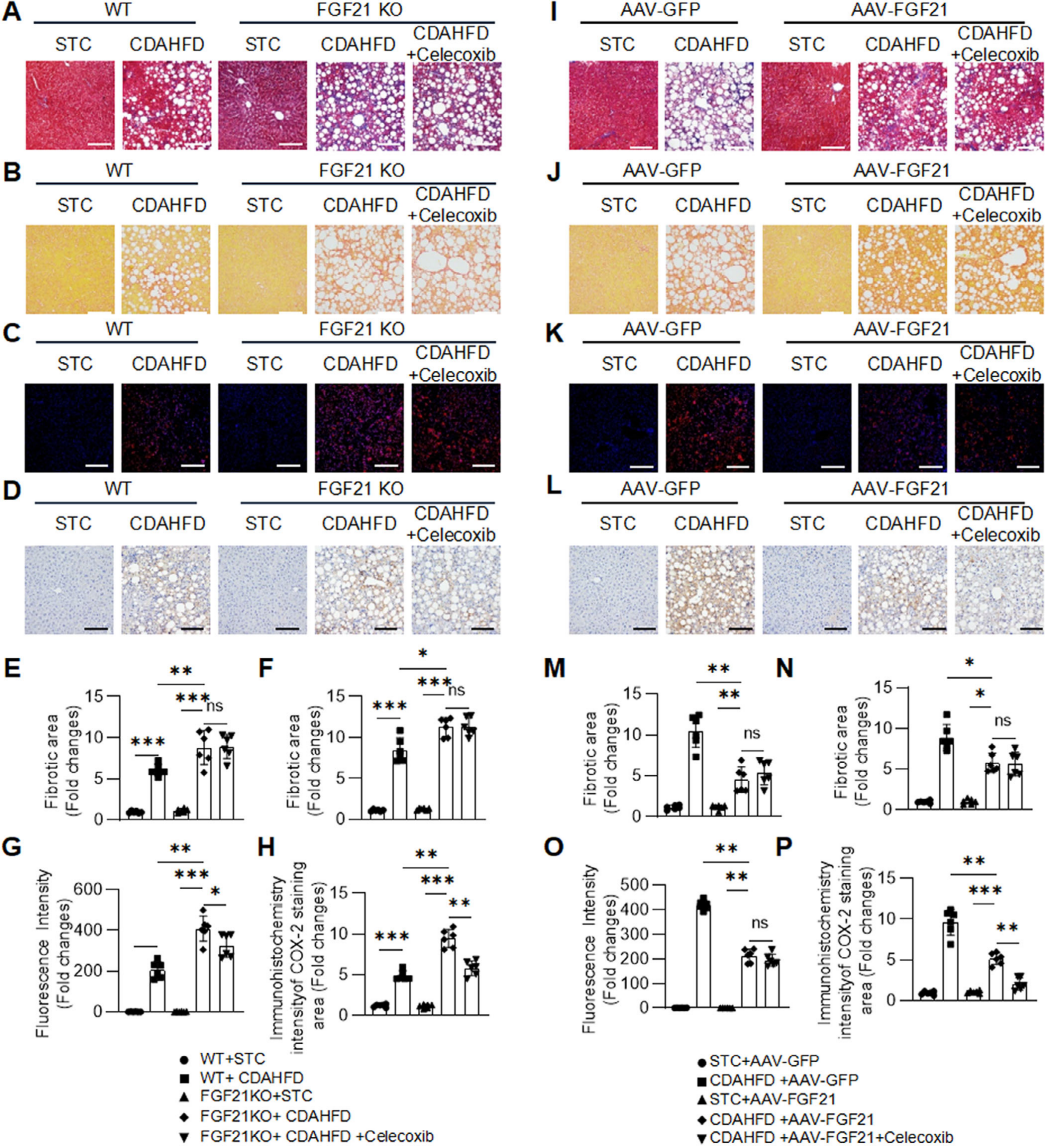
Functional validation of the FGF21‐COX‐2 axis in CDAHFD‐induced liver injury. (A) and (I) Representative Masson's trichrome staining of liver sections showing hepatic fibrosis in the indicated groups; quantification of fibrotic areas is shown in (E) and (M). Scale bar, 50 µm. (B and J) Representative Sirius Red staining showing hepatic collagen deposition in the indicated groups; quantification of Sirius Red‐positive areas is shown in (F) and (N). Scale bar, 50 µm. (C and K) Representative DHE staining assessing hepatic ROS production; quantification of fluorescence intensity is shown in (G) and (O). Scale bar, 50 µm. (D and L) Representative immunohistochemical staining for COX‐2 in the indicated groups; quantification of COX‐2‐positive staining intensity is shown in (H) and (P). Scale bar, 50 µm. Data are presented as mean ± SEM; *n* = 6 per group. Statistical comparisons among groups were performed by one‐way ANOVA followed by Tukey's post hoc test. *p < 0.05, **p < 0.01, ***p < 0.001.

To determine whether the observed effects generalize beyond HFD‐induced steatosis, we further evaluated the FGF21‐COX‐2 axis in a CDAHFD‐induced steatohepatitis model. Consistent with the findings obtained in HFD‐fed mice, FGF21 deficiency aggravated liver injury in CDAHFD‐fed mice, as evidenced by increased oxidative stress, enhanced collagen deposition, and augmented hepatic COX‐2 expression (Figure 6A–H). Additionally, FGF21 deficiency was associated with elevated serum ALT and AST levels, without marked effects on body weight, and further exacerbated hepatocyte vacuolation in CDAHFD‐fed mice (Figure S9A–D). Celecoxib treatment partially attenuated liver injury and oxidative stress but failed to reverse fibrosis in FGF21‐deficient mice. Conversely, AAV‐mediated FGF21 supplementation ameliorated CDAHFD‐induced hepatic injury and suppressed hepatic COX‐2 activation (Figure 6I–P). Consistently, AAV‐FGF21 reduced serum ALT and AST levels and alleviated hepatocyte vacuolation, whereas body weight remained largely unchanged; additional Celecoxib treatment conferred no further benefit beyond that achieved by FGF21 supplementation alone (Figure S9E–H). Collectively, these findings indicate that the contribution of the FGF21‐COX‐2 axis to MASLD progression is reproducible across distinct dietary models.

### 2.6 ERK1/2 Modulates the FGF21/PPARα Axis to Regulate COX‐2 Expression

**FIGURE 7:**
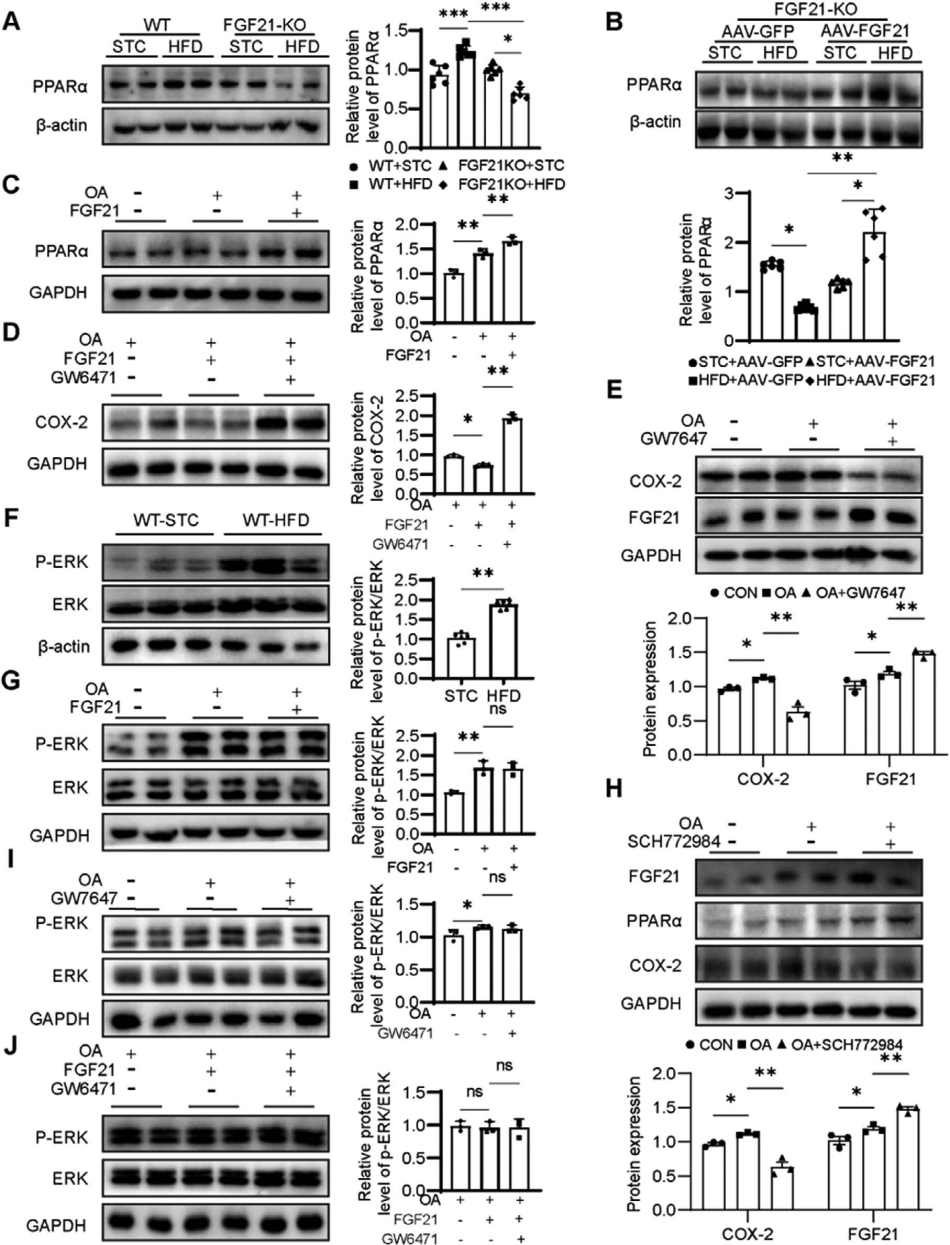
The FGF21/PPARα axis‐mediated inhibition of COX‐2 is regulated by ERK1/2 phosphorylation. Hepatic PPARα levels in FGF21KO mice (A), FGF21‐replenished mice (B), and AML12 cells (C) under the indicated treatments, examined by western blotting. (D) COX‐2 protein levels in OA‐stimulated AML12 cells treated with recombinant FGF21 in the presence or absence of the PPARα antagonist GW6471, examined by western blotting. (E) COX‐2 and FGF21 protein levels in OA‐stimulated AML12 cells treated with PPARα agonist GW7647, examined by western blotting. (F) Hepatic ERK phosphorylation in mice fed with STC or HFD, examined by western blotting. (H) FGF21, PPARα, and COX‐2 protein levels in AML12 after SCH772984 treatment, examined by western blotting. (G, I, and J) ERK phosphorylation in AML12 cells under the indicated treatments, examined by western blotting. Data are presented as mean ± SEM. For in vivo experiments (A), (B), and (F), *n* = 6 per group; statistical comparisons were performed using one‐way ANOVA followed by Tukey's post hoc test. For in vitro experiments (C–E) and (G–J), results are from three independent biological replicates; statistical analysis was performed using one‐way ANOVA followed by Tukey's post hoc test. *p < 0.05, **p < 0.01, ***p < 0.001.

Given the established interaction between FGF21 and PPARα in metabolic regulation, we next investigated whether PPARα participates in FGF21‐mediated suppression of COX‐2 expression during MASLD progression. HFD‐fed WT mice exhibited significantly elevated hepatic PPARα expression compared with STC‐fed controls, whereas FGF21 deficiency markedly attenuated this increase (Figure 7A). Conversely, AAV‐mediated FGF21 supplementation restored hepatic PPARα expression in FGF21KO mice subjected to HFD feeding (Figure 7B). Consistent with the in vivo findings, OA stimulation increased PPARα expression in AML12 cells, and recombinant FGF21 treatment further enhanced PPARα levels (Figure 7C). These findings collectively indicate that FGF21 positively regulates PPARα expression during hepatic steatosis.

To determine whether PPARα mediates the inhibitory effect of FGF21 on COX‐2 expression, OA‐treated AML12 cells were treated with recombinant FGF21 in the presence or absence of the PPARα antagonist GW6471. Pharmacological inhibition of PPARα abrogated the suppressive effect of recombinant FGF21 on COX‐2 expression (Figure 7D). Conversely, activation of PPARα by the agonist GW7647 significantly reduced COX‐2 levels while simultaneously increasing FGF21 expression (Figure 7E). These observations indicate that PPARα is required for FGF21‐mediated suppression of COX‐2 and suggest the existence of a positive regulatory loop between FGF21 and PPARα. We next explored the role of ERK1/2 signaling in regulating the FGF21/PPARα axis. HFD feeding markedly increased hepatic ERK1/2 phosphorylation in WT mice (Figure 7F). In OA‐treated AML12 cells, pharmacological inhibition of ERK1/2 phosphorylation with SCH772984 significantly elevated FGF21 and PPARα expression while reducing COX‐2 levels (Figure 7H), indicating that ERK1/2 signaling negatively regulates the FGF21/PPARα axis and thereby promotes COX‐2 expression.

To further delineate the relationship between ERK1/2 signaling and the FGF21/PPARα axis, we assessed whether recombinant FGF21 or PPARα inhibition reciprocally regulates ERK1/2 phosphorylation. Neither recombinant FGF21 nor GW6471 altered ERK1/2 phosphorylation in OA‐stimulated AML12 cells, either alone or in combination (Figure 7G,I,J). Together with the preceding observations, these findings further support a model in which ERK1/2 functions upstream of—and negatively regulates—the FGF21/PPARα axis, rather than being downstream of it.

Collectively, these results establish that ERK1/2 phosphorylation modulates the FGF21/PPARα axis and regulates COX‐2 expression. Activation of the FGF21/PPARα signaling loop suppresses COX‐2 expression and contributes to the hepatoprotective effects of FGF21 during MASLD progression.

## 3 Discussion

In this study, we identified a functional relationship between FGF21‐ and COX‐2‐mediated AA metabolism during hepatic steatosis. COX‐2 is a key enzyme in AA metabolism and plays an important role in hepatic inflammatory responses. In patients with MASLD, circulating FGF21, PGE_2_, and ALT levels were positively correlated, prompting us to further investigate the relationship between FGF21 and COX‐2 signaling. Our experimental findings showed that FGF21 deficiency enhanced hepatic COX‐2 expression and aggravated inflammation, oxidative stress, and fibrosis, whereas FGF21 restoration suppressed COX‐2 expression and ameliorated these pathological changes. Previous studies have demonstrated that COX‐2 promotes hepatic inflammation and fibrosis by increasing pro‐inflammatory cytokine production and oxidative stress [18−20]. However, the mechanisms linking metabolic regulators such as FGF21 to COX‐2 expression during hepatic steatosis remain incompletely understood. Our findings provide further insights into this relationship by showing that FGF21‐mediated suppression of COX‐2 involves PPARα signaling and is modulated by ERK1/2 phosphorylation.

Although previous studies have linked COX‐2 to inflammatory signaling cascades during hepatic steatosis [16], the role of FGF21 in modulating this process remains largely unexplored. Our findings indicate that FGF21 serves as a key regulator of hepatic inflammation, oxidative stress, and fibrosis by mitigating COX‐2‐driven inflammatory responses. This expands upon existing knowledge by providing mechanistic insights into how FGF21 modulates AA metabolism and COX‐2 signaling through its effects on PPARα. Importantly, pharmacological inhibition of COX‐2 only partially recapitulated the protective effects of FGF21. Celecoxib attenuated hepatic inflammation, oxidative stress, and liver injury in FGF21‐deficient mice but failed to reverse established fibrosis and conferred limited additional benefits following FGF21 restoration. These observations suggest that COX‐2 represents an important downstream effector, rather than the exclusive mediator, of FGF21 action in MASLD.

COX‐2 is widely implicated in inflammation and fibrosis across multiple organ systems, including the liver, whereas its functional relationship with metabolic hormones such as FGF21 remains poorly understood. Similarly, FGF21 has been studied extensively for its metabolic benefits, including lipid oxidation and glucose homeostasis, yet its influence on COX‐2‐mediated inflammatory responses during hepatic steatosis has not been fully elucidated. By establishing a link between these two molecules, our study suggests that targeting the FGF21‐PPARα‐COX‐2 axis could provide therapeutic benefits for patients with liver steatosis, particularly those with metabolic dysregulation and chronic inflammation. The incomplete rescue achieved by COX‐2 inhibition further highlights the broader hepatoprotective repertoire of FGF21 and suggests the existence of additional downstream pathways involved in fibrosis and tissue remodeling.

Oxidative stress is another key driver of hepatic inflammation and fibrosis, as excessive ROS trigger hepatocyte activation and lipid peroxidation [21, 22, 23, 24]. Our results suggest that suppression of COX‐2‐related oxidative stress contributes, at least in part, to the hepatoprotective effects of FGF21 during hepatic steatosis. In addition, emerging evidence suggests that hepatocyte senescence contributes to lipid accumulation, inflammation, and fibrosis during hepatic steatosis [25−26]. As a marker of senescence, COX‐2 has been linked to the activation of the TGF‐β1/Smad pathway, a major regulator of autophagy [27, 28]. Our study found increased TGF‐β1 expression in HFD‐fed mice, with a more pronounced elevation in α‐SMA levels in FGF21KO mice. Furthermore, Masson staining, Sirius red staining, and collagen I and III analyses revealed increased hepatic fibrosis in FGF21KO mice, whereas AAV‐FGF21 treatment reduced COX‐2 expression and ameliorated fibrosis. However, the inability of Celecoxib to reverse established fibrosis in either the HFD or CDAHFD model suggests that additional pro‐fibrotic pathways downstream of FGF21 remain to be identified. Therefore, although COX‐2 signaling contributes to fibrogenesis, it is unlikely to fully account for the anti‐fibrotic effects of FGF21.

Our metabolomic analyses further revealed that HFD markedly remodels hepatic AA metabolism, with FGF21 deficiency exacerbating these alterations, particularly within the COX pathway. Elevated hepatic PGE_2_ levels in FGF21KO mice were reversed by FGF21 replenishment, supporting the role of FGF21 in modulating AA metabolism. Previous studies have shown that PPARα activation suppresses COX‐2 via AP‐1 inhibition in colorectal cancer cells [12, 29]. Our findings extend this regulatory mechanism to liver steatosis, identifying FGF21 as a crucial modulator of this interaction. In addition, targeted assessment of hepatic PGE_2_ following pharmacological COX‐2 inhibition further validated PGE_2_ as a functional downstream readout of COX‐2 activity in the diseased liver.

Beyond COX‐2‐mediated AA metabolism, our study suggests that FGF21KO disrupts lipid metabolism, leading to excessive hepatic lipid accumulation. Notably, the liver weight‐to‐body‐weight ratio was significantly higher in FGF21KO mice, and histological analyses revealed severe hepatic steatosis and vacuolization. Liver injury markers ALT and AST were also significantly elevated in FGF21KO mice. Importantly, AAV‐FGF21 administration mitigated these metabolic disturbances, reducing lipid accumulation and liver damage. Although FGF21 restoration exerted only modest effects on systemic glucose homeostasis, its beneficial effects on hepatic inflammation, oxidative stress, fibrosis, and liver injury were substantially more pronounced. These findings suggest that the hepatoprotective actions of FGF21 cannot be solely explained by global metabolic improvements and are likely mediated, at least in part, through local suppression of the COX‐2/PGE_2_ axis.

Our study integrates in vivo and in vitro models to delineate the molecular mechanisms governing FGF21‐COX‐2 interactions. Additionally, our use of metabolomic analyses provides valuable insights into the metabolic disturbances associated with COX‐2 dysregulation during hepatic steatosis. Notably, similar observations were reproduced in the CDAHFD model, supporting the contribution of the FGF21‐COX‐2 axis across distinct stages of disease progression ranging from steatosis to steatohepatitis and fibrosis. However, given the complexity of human MASLD and its differences from murine models, validations in human liver tissues and exploration of pharmacological FGF21 analogs in clinical settings are warranted.

Despite these strengths, several limitations of the present study warrant consideration. Although our data support a functional link between the FGF21‐PPARα axis and COX‐2 regulation, direct promoter‐level regulation was not examined. Further studies are needed to investigate additional regulatory pathways involved in COX‐2 modulation by FGF21, including potential crosstalk with other metabolic and inflammatory signaling networks. In the present study, FGF21 restoration was achieved through AAV‐mediated gene delivery rather than pharmacological intervention. Therefore, whether clinically relevant FGF21 analogs, receptor agonists, or other FGF21‐based therapeutic strategies exert similar regulatory effects on the COX‐2 pathway remains to be determined. Future studies are also required to evaluate the efficacy, safety, and long‐term therapeutic potential of FGF21‐targeted interventions in chronic liver disease. Exploring the role of the FGF21‐COX‐2 axis in other metabolic disorders beyond MASLD may further broaden its translational and therapeutic significance.

In conclusion, our study identifies FGF21 as a novel upstream regulator of COX‐2 that contributes to hepatic inflammation and oxidative stress during liver steatosis progression. By elucidating the interplay between FGF21, PPARα, and COX‐2, we provide a novel perspective on the pathophysiology of MASLD and propose new potential therapeutic targets. Our findings support a model in which COX‐2 functions as an important downstream effector, but not the sole mediator, of FGF21‐dependent hepatoprotection. This work lays the foundation for future investigations into FGF21‐based interventions aimed at mitigating COX‐2‐driven inflammation and metabolic disturbances in liver disease.

## 4 Materials and Methods

### 4.1 Study Population

All participants provided written informed consent, and the study was approved by the Human Ethics Committee of Lishui People's Hospital (Approval No. 2024‐041‐01). This study enrolled a prospective cohort comprising patients with MASLD and age‐ and sex‐matched healthy controls. MASLD was diagnosed on the basis of liver biopsy or MRI‐PDFF ≥ 5%, or by hepatic steatosis detected on abdominal ultrasound (defined by at least two of the following: enhanced near‐field hepatic echoes, blurred intrahepatic vascular structures, or attenuation of far‐field echoes) or FibroScan CAP ≥ 248 dB/m. Patients were additionally required to fulfill at least one metabolic criterion: BMI ≥ 24 kg/m^2^ with increased waist circumference (≥ 90 cm in men and ≥ 85 cm in women), fasting blood glucose ≥ 5.6 mmol/L or type 2 diabetes, or triglycerides ≥ 1.7 mmol/L or low HDL‐C (< 0.9 mmol/L in men and < 1.0 mmol/L in women). Patients with viral hepatitis, autoimmune liver disease, or alcoholic liver disease (alcohol consumption ≥ 30 g/day in men or ≥ 20 g/day in women) were excluded. Healthy controls had no evidence of hepatic steatosis on ultrasound or had FibroScan CAP < 220 dB/m and were free of obesity, diabetes, or dyslipidemia. Additional exclusion criteria applied to all participants included severe cardiovascular or renal disease, malignancy, and use of medications affecting COX‐2/PGE_2_ or FGF21 signaling, vitamin E, or insulin sensitizers. A total of 54 participants (33 patients with MASLD and 21 healthy controls) were included in the final analysis.

### 4.2 Materials

The HFD (XTHF60, corresponding to D12492), the methionine‐restricted, CDAHFD (XTMRCD60, corresponding to A06071302), and their respective control diets (XTCON50J and XTMRCD10‐C) were purchased from Xietong Organism (Jiangsu, China). Celecoxib (SC 58635; MedChemExpress, Monmouth Junction, NJ, USA) was used to pharmacologically inhibit COX‐2 activity in vivo. Antibodies against COX‐2, Erk1/2, phospho‐Erk1/2, and IL‐6 were obtained from Cell Signaling Technology (Danvers, MA, USA), whereas antibodies against FGF21, TNF‐α, TGF‐β1, Collagen I, Collagen III, and α‐SMA were obtained from Abcam (Cambridge, UK). RT‐PCR kits were purchased from Thermo Fisher Scientific (Waltham, MA, USA), and assay kits for HDL, LDL, TC, and TG were obtained from Nanjing JianCheng Bioengineering Institute (Nanjing, China). H&E, Sirius Red, and Masson's trichrome staining kits were purchased from Solarbio (Beijing, China). SCH772984 was obtained from TOPSCIENCE (Shanghai, China), and GW7647 and GW6471 were obtained from Selleck Chemicals (Houston, TX, USA).

### 4.3 Animals and Diets

*fgf21* heterozygous mice were kindly donated by Professor Aimin Xu from the University of Hong Kong. Heterozygous males and females were intercrossed to generate FGF21KO mice and WT littermate controls. Mice were housed individually under specific pathogen‐free conditions at 22 ± 1°C, 60% humidity, and a 12‐h light/dark cycle. Beginning at 8 weeks of age, mice were fed either a standard control diet (10% kcal from fat) or an HFD (60% kcal from fat) for 16 weeks. In an independent validation cohort, mice received either the corresponding control diet or a CDAHFD for 8 weeks to establish a steatohepatitis‐like phenotype. All animal procedures were approved by the Animal Research Ethics Committee of Wenzhou Medical University (Approval No. xmsq2023‐1002).

### 4.4 Celecoxib Treatment

For the HFD‐induced hepatic steatosis model, mice were fed an HFD for 16 weeks, and Celecoxib was administered by oral gavage at 20 mg/kg/day during the final 8 weeks of HFD feeding. For the CDAHFD‐induced steatohepatitis model, mice were fed a CDAHFD for 8 weeks, and Celecoxib was administered at 20 mg/kg/day by oral gavage during the final 4 weeks. In the AAV‐FGF21 rescue experiments, Celecoxib treatment was initiated after stable AAV‐mediated FGF21 expression had been established and was continued until sacrifice. Control mice received an equal volume of vehicle according to the same schedule.

### 4.5 Immunoblot Analysis and Real‐Time PCR

Proteins were extracted from cells and mouse tissues, separated by sodium dodecyl sulfate‐polyacrylamide gel electrophoresis (SDS‐PAGE), and transferred onto polyvinylidene difluoride (PVDF) membranes. Horseradish peroxidase (HRP)‐conjugated rabbit anti‐IgG served as the secondary antibody. The following primary antibodies were used for immunoblotting: COX‐2 (#12282), Erk1/2 (#9102S), phospho‐Erk1/2 (#4370S), and IL‐6 (#12912) from Cell Signaling Technology; FGF21 (ab171941), TNF‐α (ab183218), Collagen I (ab138492), and α‐SMA (ab124964) from Abcam; PPARα (WL00978), SOD2 (24127‐1‐AP), HO‐1 (10701‐1‐AP), and TGF‐β1(21898‐1‐AP). Protein bands were visualized using enhanced chemiluminescence (ECL) reagents and quantified by band‐intensity analysis with ImageJ software.

Total RNA was isolated using a TRIzol reagent according to the manufacturer's protocol. Complementary DNA was synthesized, and target sequences were quantified by qRT‐PCR using SYBR Green‐based reagents. The following primer pairs were used: COX‐2 forward (5'‐TGAGCAACTATTCCAAACCAGC‐3') and reverse (5'‐GCACGTAGTCTTCGATCACTATC‐3'); EP4 forward (5'‐GCTGTCCCTGGTATCATCTGCT‐3') and reverse (5'‐AGGGTTGGGATGAAGGCTGT‐3'); TNF‐α forward (5'‐GACGTGGAACTGGCAGAAGAG‐3') and reverse (5'‐TTGGTGGTTTGTGAGTGTGAG‐3'); and IL‐6 forward (5'‐TAGTCCTTCCTACCCCAATTTCC‐3') and reverse (5'‐TTGGTCCTTAGCCACTCCTTC‐3').

### 4.6 Primary Hepatocyte Isolation and Culture

Primary hepatocytes were isolated from adult C57BL/6J WT and FGF21KO mice by two‐step collagenase perfusion. Mice were anesthetized with tribromoethanol dissolved in saline (0.2–0.3 mL), and livers were sequentially perfused through the inferior vena cava with PBS, PBS containing EGTA, and 0.25 mg/mL collagenase type IV. Excised livers were further digested with 0.5 mg/mL collagenase type IV, and digestion was terminated with DMEM containing 10% serum and 1 g/L glucose. The cell suspension was filtered through a 70‐µm cell strainer and purified by repeated low‐speed centrifugation (80 g, 1 min). Hepatocytes were resuspended in DMEM containing 10% serum and 1 g/L glucose and plated in six‐well plates or culture dishes. The medium was replaced after 30 min, and subsequent treatments were performed after 12 h of starvation.

### 4.7 Targeted Oxylipidomic Analysis

Serum and liver samples were collected at sacrifice and stored at −80°C until analysis. Samples were extracted with methanol/acetonitrile (1:1, v/v) containing deuterated internal standards and purified by C18 solid‐phase extraction. Chromatographic separation was performed on a Waters ACQUITY UPLC HSS T3 C18 column (1.8 µm, 2.1 × 100 mm, 40°C) using gradient elution with acetonitrile/water and acetonitrile/isopropanol. Targeted oxylipidomic analysis was conducted on a SCIEX QTRAP 6500+ UPLC‐MS/MS system in negative electrospray ionization and multiple reaction monitoring modes. Metabolites were quantified using external calibration with authentic standards, and data were processed with MultiQuant 3.0.3 software.

### 4.8 Glucose Tolerance Test and Insulin Tolerance Test

Glucose tolerance and insulin sensitivity were evaluated by intraperitoneal glucose tolerance tests (IGTT) and insulin tolerance tests (ITT), respectively. For IGTT, mice were fasted for 6 h with free access to water and then received an intraperitoneal injection of glucose at 2 g/kg body weight. Blood glucose was measured from the tail vein using a handheld glucometer at 0, 15, 30, 60, 90, and 120 min after glucose administration, and the area under the curve (AUC) was calculated. For the ITT, mice were fasted for 4 h and intraperitoneally injected with insulin at 0.75 U/kg body weight. Tail‐vein blood glucose was measured at 0, 15, 30, 45, and 60 min, and the AUC was calculated to assess systemic insulin sensitivity.

### 4.9 Metabolic Cage Analysis

Whole‐body energy metabolism was assessed using a PhenoMaster metabolic monitoring system (TSE Systems, Germany) at the Experimental Animal Center of Wenzhou Medical University. Mice were individually housed in metabolic cages under controlled conditions with a 12‐h light/dark cycle and free access to food and water. After a 24‐h acclimatization period, metabolic parameters were recorded continuously for an additional 24 h. Food intake, oxygen consumption (VO_2_), carbon dioxide production (VCO_2_), and respiratory exchange ratio (RER) were automatically measured and analyzed with PhenoMaster software according to the manufacturer's instructions.

### 4.10 Body Composition Analysis

Body composition was assessed using a small‐animal body composition analyzer (EchoMRI LLC, Houston, TX, USA) at the Experimental Animal Center of Wenzhou Medical University. Conscious mice were placed individually into the instrument according to the manufacturer's instructions. Body fat percentage was automatically determined and analyzed by the accompanying software. Measurements were performed at the end of the dietary intervention prior to sacrifice.

### 4.11 Histological Analysis

Liver tissue from corresponding anatomical sites was excised and fixed in 4% buffered paraformaldehyde overnight, then embedded in paraffin. Sections (5 µm) were cut and subjected to H&E, Masson, and Sirius red staining according to standard protocols.

### 4.12 Immunohistochemistry (IHC)

Immunohistochemistry was performed to assess COX‐2, α‐SMA, and TNF‐α expression. Paraffin‐embedded sections were deparaffinized, rehydrated, and subjected to antigen retrieval in 10 mM sodium citrate buffer. After blocking endogenous peroxidase activity and nonspecific binding, sections were incubated overnight at 4°C with the indicated primary antibodies, followed by an HRP‐conjugated secondary antibody. Signals were visualized with 3,3′‐diaminobenzidine and counterstained with hematoxylin.

### 4.13 Statistical Analysis

All statistical analyses were performed using GraphPad Prism 8 software (GraphPad Software, San Diego, CA, USA). Data are presented as mean ± SEM unless otherwise specified. Comparison between two groups were analyzed by unpaired two‐tailed Student's *t*‐test. Comparisons among multiple groups were performed by one‐way analysis of variance (ANOVA), followed by Tukey's post hoc test when appropriate. Correlations were assessed by Pearson's correlation analysis. A *p* value < 0.05 was considered statistically significant.

## Author Contributions

Guangsen Xu, Tingting Pan, and Guiyun Wang contributed to data collection and literature review, and wrote the first draft of the manuscript. Junlin Leng, Feng Qiu, Qiyang Feng, Yongguo Xue, Ye Wang, Junwei Zheng, Huiying Lu, Bingyan Han, and Suntao Wang contributed to data collection, and performed statistical analysis. Jie Du provided funding support for the study. Jiaojian Lv provided clinical samples and contributed to conception of the study and funding support. Xuebo Pan and Baile Wang contributed to conception and design of the study, funding acquisition, and revision of the manuscript. All authors read and approved the final manuscript.

## Funding

This work was funded by the Natural Science Foundation of Zhejiang Province (Grant Number: LR20H310001), Zhejiang Provincial Medical and Health Technology Planning Project (2022KY1444); Basic Research Operating Fee of Wenzhou Medical University: Regulation of Arachidonic Acid Metabolism by FGF21 and Its Role in Non‐Alcoholic Fatty Liver Lesions (KYYW202004), and Science and Technology Program of Wenzhou (Grant/Award Number: ZY2021010).

## Ethics Statement

The human study was conducted in accordance with the Declaration of Helsinki and was approved by the Human Ethics Committee of Lishui People's Hospital (Approval No. 2024‐041‐01). Written informed consent was obtained from all participants prior to enrollment. All animal experiments were performed in accordance with the National Institutes of Health Guide for the Care and Use of Laboratory Animals and were approved by the Animal Research Ethics Committee of Wenzhou Medical University (Approval No. xmsq2023‐1002).

## Conflicts of Interest

The authors declare no conflicts of interest.

## Supporting information




**Supporting file**: mco270999‐sup‐0001‐SuppMat.docx.

## Data Availability

The data supporting the findings of this study are available from the corresponding author upon reasonable request. Data generated during the current study have been deposited in the Zenodo repository: https://doi.org/10.5281/zenodo.20765975.
