# Supplementary material for: Inhibition of COX‐2‐Mediated Arachidonic Acid Metabolism by FGF21‐PPARα Axis Alleviates Liver Steatosis
Source: MedComm (2020). 2026 Sep 15;7(10):e70999. doi: 10.1002/mco2.70999 (PMC13575766; doi:10.1002/mco2.70999)
Supplement: Supplementary file 1 [file MCO2-7-e70999-s001.docx]

**Inhibition of COX-2-mediated arachidonic acid metabolism by FGF21-PPARα axis alleviates liver steatosis**

**Guangsen Xu^1#^**, **Tingting Pan^1,6#^**, **Guiyun Wang^1#^**, Feng Qiu^1,7^, Junlin Leng^3,4,5^, Qiyang Feng^1^, Yongguo Xue^1^, Ye Wang^1^, Junwei Zheng^1^, Huiying Lu^1^, Bingyan Han^1^, Suntao Wang^1^, Jie Du ^8^, Jiaojian Lv^2^*, Baile Wang^3,4,5^*, Xuebo Pan^1^*

^1^ School of Pharmaceutical Sciences, Wenzhou Medical University, University Town, Wenzhou, Zhejiang Province, 325035, China

^2^ Department of Infectious Diseases, The sixth affiliated hospital of Wenzhou medical university, Lishui People's Hospital, Lishui, Zhejiang Province, 323000, China

^3^ State Key Laboratory of Pharmaceutical Biotechnology, The University of Hong Kong, Hong Kong, China

^4^ Department of Medicine, The University of Hong Kong, Hong Kong, China

^5^ Guangdong-Hong Kong Joint Laboratory for Metabolic Medicine, The University of Hong Kong, Hong Kong, China

^6^ Taizhou traditional Chinese medicine hospital, Taizhou, Zhejiang Province, 318000, China

^7^ Department of Genetics and Developmental Science, School of Life Sciences and Biotechnology, Shanghai Jiao Tong University, Shanghai, China

^8^ Department of Cardiothoracic Surgery, The Second Affiliated Hospital of Wenzhou Medical University, 109 West Xue Yuan Road，Wenzhou，Zhejiang, 325000, China

\# These authors contributed equally to this work.

* Corresponding authors:

[xuebopan@wmu.edu.cn](mailto:xuebopan@wmu.edu.cn); baile612@hku.hk; [lslvjiaojian@126.com](mailto:lslvjiaojian@126.com);

**Supplementary Table 1. Sociodemographic and clinical characteristics of the 54 subjects according to Healthy controls vs MASLD.**

|  | **Healthy controls** | **MASLD** | **P value** |
| --- | --- | --- | --- |
| **Sample size n (%)** | 21(38.89%) | 33(61.11%) |  |
| **Age, in years, mean±SD** | 46.00 ± 10.19 | 49.48 ± 14.62 | 0.4630 |
| **BMI, mean ± SD (kg/m2)** | 21.95 ± 2.48 | 26.35 ± 3.58 | <0.0001 |
| **Total cholesterol, mean ± SD (mmol/L)** | 4.66 ± 0.66 | 5.56 ± 1.05 | 0.0005 |
| **High-density lipoprotein, mean ± SD (mmol/L)** | 1.37 ± 0.28 | 1.30 ± 1.05 | 0.4369 |
| **Low-density lipoprotein, mean ± SD (mmol/L)** | 2.60 ± 0.60 | 3.04 ± 0.51 | 0.0043 |
| **Triglycerides (mmol/L)** | 1.18 ± 0.60 | 2.41 ± 1.56 | 0.0006 |
| **Fasting plasma glucose, mean ± SD (mmol/L)** | 5.07 ± 0.57 | 6.10 ± 1.64 | 0.0050 |
| **Controlled attenuation parameter, mean ± SD (dB/m)** | 205.71 ± 21.49 | 299.79 ± 40.14 | <0.0001 |
| **Liver stiffness measurement, mean ± SD (KPa)** | 5.05 ± 1.05 | 8.02 ± 3.58 | 0.0002 |

**Supplementary Figure 1. Associations among circulating FGF21, PGE_2_, and liver injury after adjustment for BMI and hepatic steatosis**. (A) Partial correlation analysis between serum FGF21 levels and serum ALT levels after adjustment for body mass index (BMI) and controlled attenuation parameter (CAP); (B) Partial correlation analysis between serum PGE_2_ levels and serum ALT levels after adjustment for BMI and CAP; (C) Partial correlation analysis between serum FGF21 and serum PGE_2_ levels after adjustment for BMI and CAP. The red line represents the fitted linear regression line, and the shaded area indicates the 95% confidence interval. Circles represent patients with MASLD, whereas triangles represent healthy controls. Partial correlation coefficients (partial *r*) and corresponding *P* values are shown in each panel. Statistical significance was assessed using partial correlation analysis controlling for BMI and CAP.

**Supplementary****Figure 2. FGF21 deficiency exacerbates HFD-induced hepatic steatosis and inflammatory responses without markedly altering body weight gain.** (A) Body weight trajectories of WT+STC, WT+HFD, FGF21KO+STC, and FGF21KO+HFD mice during the 16-week dietary intervention; (B) Body fat percentage in the indicated groups; (C) Representative gross morphology of liver tissues from the indicated groups. Scale bar, 1 cm; (D) Tissue weight ratios of liver, subcutaneous adipose tissue (SAT), white adipose tissue (WAT), and brown adipose tissue (BAT) in the indicated groups; (E) Representative hematoxylin and eosin (H&E) staining of liver sections showing hepatic steatosis. Quantification of the total area of hepatocyte vacuolation is shown. Scale bar, 50 μm; (F) and (G) Relative mRNA levels of IL-6 and TNF-α in liver tissues determined by real-time quantitative PCR, respectively; (H) and (I) Representative images of liver tissues with immunohistochemical staining for α-SMA and TNF-α, respectively. Quantification of the positive staining area is shown. Scale bar, 50 μm. Data are expressed as mean ± SEM; n=6 per group; Statistical comparisons among groups were performed using one-way ANOVA followed by Tukey’s post hoc test; * P<0.05, ** P<0.01, *** P<0.001.

**Supplementary Figure 3. Metabolic characterization of WT and FGF21KO mice under STC and HFD conditions.** (A) and (B) Food consumption of mice in grams over 24 hours; (C) and (D) Oxygen consumption (VO_2_) normalized to body weight over 24 h monitored by a metabolic cage; (E) and (F) Carbon dioxide production (VCO_2_) normalized to body weight over 24 h monitored by a metabolic cage; (G) and (H) Respiratory exchange ratio (RER) calculated from data given in C-F. Data are expressed as mean ± SEM; n=6 per group; Statistical comparisons among groups were performed using one-way ANOVA followed by Tukey’s post hoc test; * P<0.05, ** P<0.01, *** P<0.001.

**Supplementary Figure 4.** **Systemic metabolic and biochemical characterization of FGF21-deficient and FGF21-restored mice under HFD conditions.** (A-D) Serum levels of total cholesterol (TC), triglycerides (TG), low-density lipoprotein cholesterol (LDL-C), and high-density lipoprotein cholesterol (HDL-C) in WT+STC, WT+HFD, FGF21KO+STC, and FGF21KO+HFD mice; (E) and (F) Glucose tolerance test (GTT) and insulin tolerance test (ITT), respectively, in the indicated groups. Quantification of the area under the curve (AUC) is shown; (G) and (H) Serum alanine aminotransferase (ALT) and aspartate aminotransferase (AST) levels in the indicated groups; (I-L) Serum levels of TC, TG, LDL-C, and HDL-C in FGF21KO+STC+AAV-GFP, FGF21KO+HFD+AAV-GFP, FGF21KO+STC+AAV-FGF21, and FGF21KO+HFD+AAV-FGF21 mice; (M) and (N) GTT and ITT, respectively, in the indicated groups. Quantification of the AUC is shown; (O) and (P) Serum ALT and AST levels in the indicated groups. Data are expressed as mean ± SEM; n=6 per group; Statistical comparisons among groups were performed using one-way ANOVA followed by Tukey’s post hoc test; * P<0.05, ** P<0.01, *** P<0.001.

**Supplementary Figure 5. Serum arachidonic acid metabolites in WT and FGF21KO mice under STC and HFD conditions**. Selected products on the AA metabolic pathway, including the P450 pathway (20-HETE, 5,6-EET, 11,12-EET, and 14,15-EET), LOX pathway (15-HETE, LTB_4_, 12-HETE and 5-HETE) and the COX pathway (PGE_2_, PGD_2_, PGF_2_α, PGI_2_, 15d-PGJ_2_, and PGFM). Data are expressed as mean ± SEM; n=6 per group; Statistical comparisons among groups were performed using one-way ANOVA followed by Tukey’s post hoc test; * P<0.05, ** P<0.01, *** P<0.001.

**Supplementary****Figure 6. FGF21 restoration ameliorates HFD-induced systemic and hepatic abnormalities in FGF21KO mice.** (A) Body weight trajectories of FGF21KO+STC+AAV-GFP, FGF21KO+HFD+AAV-GFP, FGF21KO+STC+AAV-FGF21, and FGF21KO+HFD+AAV-FGF21 mice during the 16-week dietary intervention; (B) Body fat percentage in the indicated groups; (C) Tissue weight ratios of liver, subcutaneous adipose tissue (SAT), white adipose tissue (WAT), and brown adipose tissue (BAT) in the indicated groups; (D) Representative gross morphology of liver tissues from the indicated groups. Scale bar, 1 cm; (E) Representative hematoxylin and eosin (H&E) staining of liver sections showing hepatic steatosis. Quantification of the total area of hepatocyte vacuolation is shown. Scale bar, 50 μm; (F) and (G) Relative mRNA levels of IL-6 and TNF-α in liver tissues determined by real-time quantitative PCR, respectively; (H) Serum FGF21 concentrations in the indicated groups determined by ELISA. Data are expressed as mean ± SEM; n=6 per group; Statistical comparisons among groups were performed using one-way ANOVA followed by Tukey’s post hoc test; * P<0.05, ** P<0.01, *** P<0.001.

**Supplementary Figure 7. FGF21 reconstitution normalizes hepatic arachidonic acid metabolism in FGF21KO mice under STC and HFD conditions.** Hepatic levels of specific products on the three AA metabolic pathways of indicated mice measured by LC-MS/MS, namely, 5,6-EET, 8,9-EET, 11,12-EET, and 14,15-EET of the P450 pathway; 15-HETE, 12-HETE and 5-HETE of LOX pathway; and PGD_2_, PGF_2_α, PGE_2_, and 15d-PGJ_2_ of COX pathway. Data are expressed as mean ± SEM; n=6 per group; Statistical comparisons among groups were performed using one-way ANOVA followed by Tukey’s post hoc test; * P<0.05, ** P<0.01, *** P<0.001.

**Supplementary****Figure 8. Effects of Celecoxib treatment on metabolic and hepatic phenotypes in FGF21-deficient mice and FGF21-restored mice under HFD conditions.** (A) Representative gross morphology of liver tissues from WT+HFD, FGF21KO+HFD, and FGF21KO+HFD+Celecoxib mice; (B) Representative hematoxylin and eosin (H&E) staining of liver sections from the indicated groups. Quantification of the total area of hepatocyte vacuoles is shown. Scale bar, 50 μm; (C) Body weight trajectories of WT+HFD, FGF21KO+HFD, and FGF21KO+HFD+Celecoxib mice during the 16-week dietary intervention; (D) Body fat percentage in the indicated groups; (E) Hepatic PGE₂ concentrations in the indicated groups determined by ELISA; (F) and (G) Serum alanine aminotransferase (ALT) and aspartate aminotransferase (AST) levels in the indicated groups; (H) Representative gross morphology of liver tissues from FGF21KO+HFD+AAV-GFP, FGF21KO+HFD+AAV-FGF21, and FGF21KO+HFD+AAV-FGF21+Celecoxib mice; (I) Representative H&E staining of liver sections from the indicated groups. Quantification of the total area of hepatocyte vacuoles is shown. Scale bar, 50 μm; (J) Body weight trajectories of FGF21KO+HFD+AAV-GFP, FGF21KO+HFD+AAV-FGF21, and FGF21KO+HFD+AAV-FGF21+Celecoxib mice during the 16-week dietary intervention; (K) Body fat percentage in the indicated groups; (L) Hepatic PGE₂ concentrations in the indicated groups determined by ELISA; (M) and (N) Serum ALT and AST levels in the indicated groups. Data are expressed as mean ± SEM; n=6 per group; Statistical comparisons among groups were performed using one-way ANOVA followed by Tukey’s post hoc test; * P<0.05, ** P<0.01, *** P<0.001.

**Supplementary****Figure 9.** **Additional metabolic and liver injury phenotyping in CDAHFD-fed mice subjected to FGF21 deficiency, FGF21 restoration, and COX-2 inhibition.** (A) and (B) Serum alanine aminotransferase (ALT) and aspartate aminotransferase (AST) levels in WT+STC, WT+CDAHFD, FGF21KO+STC, FGF21KO+CDAHFD, and FGF21KO+CDAHFD+Celecoxib mice; (C) Body weight trajectories of the indicated groups during the 8-week CDAHFD intervention; (D) Representative hematoxylin and eosin (H&E) staining of liver sections from the indicated groups. Quantification of the total area of hepatocyte vacuoles is shown. Scale bar, 50 μm; (E) and (F) Serum alanine aminotransferase (ALT) and aspartate aminotransferase (AST) levels in FGF21KO+CDAHFD+AAV-GFP, FGF21KO+CDAHFD+AAV-FGF21, and FGF21KO+CDAHFD+AAV-FGF21+Celecoxib mice, together with their respective STC controls; (G) Body weight trajectories of the indicated groups during the 8-week CDAHFD intervention; (H) Representative hematoxylin and eosin (H&E) staining of liver sections from the indicated groups. Quantification of the total area of hepatocyte vacuoles is shown. Scale bar, 50 μm. Data are expressed as mean ± SEM; n=6 per group; Statistical comparisons among groups were performed using one-way ANOVA followed by Tukey’s post hoc test; * P<0.05, ** P<0.01, *** P<0.001.

**Supplementary Figure 10. Proposed model illustrating the role of the FGF21-PPARα-COX-2 axis in regulating arachidonic acid metabolism during steatotic liver disease.** Under physiological conditions, the FGF21-PPARα axis restrains COX-2 activity and maintains arachidonic acid (AA) metabolic homeostasis. In steatotic liver disease induced by OA, HFD, or CDAHFD, ERK1/2 phosphorylation is activated, while FGF21 and PPARα are adaptively upregulated. However, this adaptive response is insufficient to fully suppress COX-2 activation, resulting in enhanced COX-driven AA metabolic reprogramming and increased production of prostaglandin E₂ (PGE₂). Elevated PGE₂ signaling contributes to hepatic inflammation and oxidative stress, thereby promoting liver injury. Inhibition of ERK1/2 phosphorylation activates the FGF21-PPARα axis and suppresses COX-2-mediated AA metabolic remodeling.
